# Economic analysis of digital motor rehabilitation technologies: a systematic review

**DOI:** 10.1186/s13561-024-00523-5

**Published:** 2024-07-17

**Authors:** Koffi Adzinyo Agbemanyole, Kokouvi Geovani Agbohessou, Christelle Pons, Philippe Lenca, Olivier Rémy-Néris, Myriam Le Goff-Pronost

**Affiliations:** 1 IMT Atlantique, LaTIM, UMR INSERM 1101, F-29238 Brest, France; 2 IMT Atlantique, Lab-STICC, UMR CNRS 6285, F-29238 Brest, France; 3 Univ. Limoges, HAVAE, UR20217, F-87000 Limoges, France; 4 LaTIM (Laboratory of Medical Information Processing), INSERM UMR 1101 (Institut National de la Santé et de la Recherche Médicale, Unité Mixte de Recherche), 29238 Brest, France; 5 Physical Medicine and Rehabilitation Department, CHU de Brest, Hôpital Morvan, 29200 Brest, France; 6 UFR (Unité de Formation et de Recherche) Médecine, University of Western Brittany (UBO), 29238 Brest, France; 7 Pediatric Rehabilitation Department, Fondation Ildys, 29200 Brest, France

**Keywords:** Cost-effectiveness analysis, Disability, Rehabilitation technologies, QALY, Systematic review

## Abstract

Rehabilitation technologies offer promising opportunities for interventions for patients with motor disabilities. However, their use in routine care remains limited due to their high cost and persistent doubts about their cost-effectiveness. Providing solid evidence of the economic efficiency of rehabilitation technologies would help dispel these doubts in order to better take advantage of these technologies. In this context, this systematic review aimed to examine the cost-effectiveness of rehabilitation interventions based on the use of digital technologies. In total, 660 articles published between 2011 and 2021 were identified, of which eleven studies met all the inclusion criteria. Of these eleven studies, seven proved to be cost-effective, while four were not. Four studies used cost-utility analyses (CUAs) and seven used cost-minimization analyses (CMAs). The majority (ten studies) focused on the rehabilitation of the upper and/or lower limbs after a stroke, while only one study examined the rehabilitation of the lower limbs after knee arthroplasty. Regarding the evaluated devices, seven studies analyzed the cost-effectiveness of robotic rehabilitation and four analyzed rehabilitation with virtual reality.The assessment of the quality of the included studies using the CHEERS (Consolidated Health Economic Evaluation Reporting Standards) suggested that the quality was related to the economic analysis method: all studies that adopted a cost-utility analysis obtained a high quality score (above 80%), while the quality scores of the cost-minimization analyses were average, with the highest score obtained by a CMA being 72%. The average quality score of all the articles was 75%, ranging between 52 and 100. Of the four studies with a considering score, two concluded that there was equivalence between the intervention and conventional care in terms of cost-effectiveness, one concluded that the intervention dominated, while the last one concluded that usual care dominated. This suggests that even considering the quality of the included studies, rehabilitation interventions based on digital technologies remain cost-effective, they improved health outcomes and quality of life for patients with motor disorders while also allowing cost savings.

**Supplementary Information:**

The online version contains supplementary material available at 10.1186/s13561-024-00523-5.

## Introduction

Rehabilitation is defined as "a set of interventions designed to optimize functioning and reduce disability in individuals with health problems interacting with their environment" [6]. Rehabilitation interventions aim to help an individual overcome difficulty in thinking, seeing, hearing, communicating, eating, or moving [37].

To be effective, motor rehabilitation programs must adhere to the principles of motor learning, i.e., they must be early, intense, multidisciplinary, task-oriented, and provide feedback to the patient [38]. However, several obstacles to adhering to these principles have been identified, including individual^1^, social^2^, and environmental challenges^3^ as well as challenges related to rehabilitation personnel^4^ [35].

Rehabilitation assisted by digital technologies clearly emerges as a solution to support healthcare professionals by providing high-intensity, repetitive, and task-specific exercises with the aim of improving the rehabilitation process [23]. The high costs associated with digital rehabilitation technologies pose a barrier to their adoption in routine rehabilitation care. In the economic literature, evidence of the cost-effectiveness of these technologies has sparked intense debate and lacks consensus. Some systematic reviews have been conducted to evaluate the consumption of healthcare resources, costs, or overall cost-effectiveness of rehabilitation technologies [20, 24]. These reviews suggested that rehabilitation technologies (RT) can be effective in improving clinical outcomes and reducing healthcare costs for people with physical disabilities. In recent years, there has been growing interest in the cost-effectiveness of digital technologies, particularly virtual reality (VR) tools and video games, as well as robotic technologies in the field of motor rehabilitation [9, 21, 31]. This systematic review aimed to synthesize the most recent evidence on the cost-effectiveness of digital motor rehabilitation technologies compared to conventional care, considering the latest studies and methodological guidelines for economic evaluations, in order to inform decision-making regarding the adoption of these often-costly technologies in routine rehabilitation care.

## Methods

This review was conducted following the Preferred Reporting Items for Systematic Reviews and Meta-Analyses (PRISMA) guidelines [28] and its associated checklist (see Appendix 1). The review protocol was registered on the International Prospective Register of Systematic Reviews (PROSPERO) under registration number CRD42023448095.

### Study selection

#### Inclusion criteria

- ▶ Types of studies

We included randomised controlled trials (RCTs).

- ▶ Types of interventions

Studies evaluating motor rehabilitation interventions based on digital technologies (DTs) such as robot-assisted gait therapy, virtual reality, augmented reality, and telerehabilitation were included. There were no restrictions on the type of DT used.

- ▶ Types of economic evaluation methods

For this systematic review, we included economic evaluations that employed one of the four methods used in healthcare to assess the cost-effectiveness of interventions: cost-effectiveness analysis (CEA), cost-utility analysis (CUA), cost-benefit analysis (CBA), and cost-minimization analysis (CMA). For detailed descriptions of these approaches, please refer to the Appendix.

##### Types of participants

Individuals aged 18 or older with motor disabilities, regardless of the underlying condition that caused it.

##### Language of publication

Only studies published in English or French were included in this systematic review. These inclusion criteria are presented in in Appendix 2.

#### Exclusion criteria

Studies that did not evaluate motor rehabilitation assisted by digital technologies or that were not full economic evaluations in health care were excluded. Reports that were not full articles, such as expert opinions, protocols, narrative reviews, treatment guidelines, and recommendations, were also excluded.

#### Search terms

A search strategy tailored to the electronic databases PubMed, Web of Science, Science Direct, Scopus, and the Cochrane Library (CENTRAL) was developed with the assistance of a methodologist. Keywords and Medical Subject Headings (MeSH) terms related to motor impairment, rehabilitation, digital rehabilitation technologies, and economic evaluation were used to formulate the PubMed query. This query was then adapted for use in other databases. The queries employed in the different databases are presented in appendixes 3 and 4. Initial searches were conducted on these databases in May 2023, with an update in May 2024, to identify studies published over the past two decades and those that have employed the most recent economic evaluation methodologies.

#### Study selection

 The examination of the identified articles took place in three stages. First, the titles and abstracts were reviewed to select eligible articles. Then, the full texts of the articles retained from the first stage were examined to determine their final inclusion. Finally, the relevant data from the included articles were extracted.

The initial selection of studies was conducted by assessing titles and abstracts retrieved through database queries. After removing duplicates on the Rayyan platform [27], two researchers (K.A.A. and K.G.A.) independently reviewed the titles and abstracts to exclude studies deemed irrelevant according to the inclusion criteria. The full texts of the selected articles were then read to determine their eligibility. Disagreements were resolved through consensus with a third researcher (M.L.G.P). The percentage agreement between the two authors regarding the included studies was estimated.

Out of the 660 records initially identified from the databases, 563 were screened after removing duplicates (see PRISMA diagram). Following the examination of titles and abstracts, 543 were excluded due to lack of relevance. Twenty articles underwent full-text review, of which 9 were excluded for the following reasons: 5 [2, 3, 19, 24, 29] for an inadequate study design, 3 [10, 15] for an intervention not involving rehabilitation technologies, and 1 [32] for a non-conventional comparator. Overall, 11 articles meeting the inclusion criteria were included in the review. The percentage agreement between the two reviewers was 97.42% (529/543 articles).

**Fig. 1:**
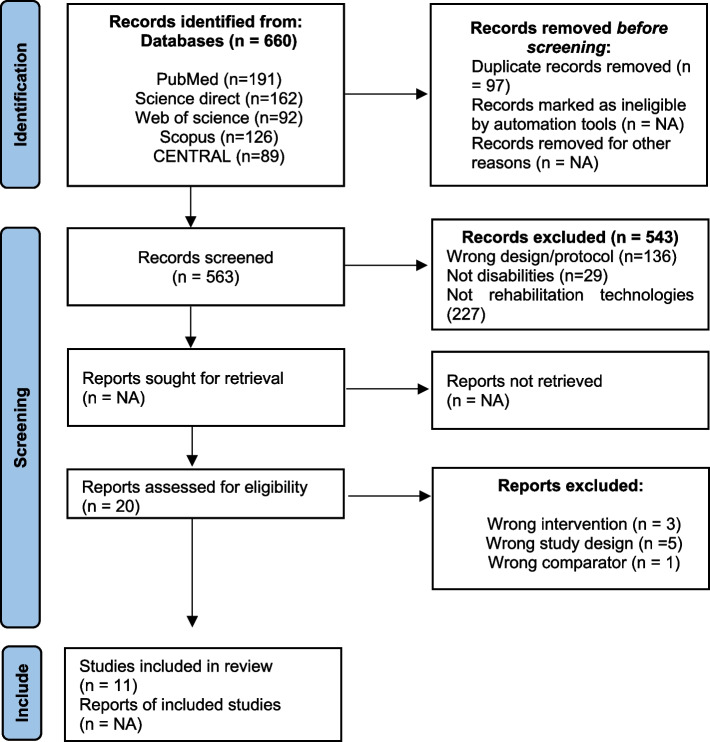
Study selection process for this systematic review (PRISMA)

The study inclusion process is summarized in Fig. 1.

#### Data extraction

To enable data comparison, data were extracted using a standardized form by the two researchers. Information regarding the included studies (year of publication, country, study design), patient data, temporal horizon, study perspective, discounting, details on the intervention and control group, evaluation of outcomes and costs, incremental costs and incremental outcomes, incremental cost-effectiveness ratio (ICER), and sensitivity analyses were extracted. The extracted data were then subjected to synthesis, analysis, and a narrative summary by researcher K.A.A. Researcher K.G.A., on the other hand, was responsible for ensuring the relevance of the entered data in accordance with the study's objective.

#### Quality assessment

The methodological quality of the included studies was assessed by two researchers using the Consolidated Health Economic Evaluation Reporting Scale (CHEERS) checklist [17], the 2022 version of which comprises 28 items presented in the Appendix. Each item on the CHEERS checklist received a score of 1 if identified (YES) in the study, 0.5 if partially identified (YES*), 0 if not identified (NO), and NA if not applicable. All the articles were assigned an overall quality score. A high score indicates high-quality reporting. In general, quality scores ranging between 10 and 30, 40 and 70, and 80 and 100 out of 100 are indicative of a low-quality article, a moderate-quality article, and a high-quality article, respectively [7].

#### Data synthesis

First, we presented the results of article selection and the characteristics of the populations studied in the included studies. Next, we addressed the foundational choices of economic evaluation within the included studies [26]. Subsequently, we presented the results of the methodological quality assessment, which is crucial for the validity and reliability of the conclusions of this systematic review. These scores are expressed as percentages based on the CHEERS checklist. Finally, in the last section devoted to cost-effectiveness results, we present the findings of all included studies, followed by results categorized by types of digital rehabilitation technologies. A dominance analysis was carried out using a dominance ranking matrix (DRM) [11]. The dominance ranking matrix (DRM) is a classification tool developed by the Joanna Briggs Institute (JBI) to interpret economic evaluation results in systematic reviews. The DRM is a three-by-three matrix with the following options:

(i) ***Strong dominance***: When the intervention is more effective and less costly, equally effective and less costly, or more effective with equal or lower cost, it is favored for efficiency.
(ii) ***Weak dominance***: When the intervention is equally effective and costly, more effective but costlier, or less effective but less costly, no conclusion is drawn about its preference without considering decision makers' priorities.
(iii) **Nondominance**: When the intervention is costlier and less effective, equally costly but less effective, or costlier but equally effective, this suggests that the comparator is favorable for efficiency.

The analysis is presented in the form of a permutation matrix showing the 9 results that exist in terms of cost-effectiveness. This matrix allows for classifying studies into 3 bands: dominance of intervention, dominance of control, and intervention equivalent to control. The presence of a certain number of studies in one of the bands allows us to draw conclusions about the cost-effectiveness of the intervention.

## Results

### Settings and population

The 11 included studies were published over the last 15 years, between 2011 and 2021. Significant heterogeneity is observed among these studies in terms of countries, characteristics of the studied populations, evaluated interventions, and objectives, reflecting the diversity of contexts and approaches for the economic evaluation of digital motor rehabilitation technologies.

Indeed, of the eleven included studies, four were conducted in the USA [16, 30, 34, 36], two in the UK [1, 9], one in France [31], one in Germany [13], one in Spain [22], and one in Mexico [4]. The eleventh study [18] was an international trial conducted in three European countries: Belgium, Norway, and Denmark. The articles were published over the past two decades, between 2011 and 2021, but the majority (10/11) were published within the last 10 years.

There is important heterogeneity among the interventions in the trials included in the review. Most studies compared the intervention group to a group receiving conventional therapy, except for two studies that used three comparison arms, with two arms receiving interventions based on rehabilitation technology and one arm receiving conventional therapy [9, 36].

Seven studies examined robot-assisted rehabilitation interventions. Among these studies, four [13, 31, 34, 36] involved rehabilitation sessions in a specialized center, while another focused on home-based rehabilitation through tele-rehabilitation [16]. Additionally, two studies evaluated group rehabilitation in a gym setting.

Four studies examined rehabilitation interventions using virtual reality technology, whether at the patient's home [30] or in a rehabilitation center [1, 18, 22].

Seven trials addressed upper limb rehabilitation following upper limb motor impairment after stroke. Among them, five [9, 13, 31, 34, 36] used robotic technology, and the other two used virtual reality [1, 18]. Two studies [4, 16] evaluated both upper and lower limb rehabilitation with robotic technology. Finally, two studies [2, 30] assessed lower limb rehabilitation using virtual reality technology.

Ten studies focused on stroke, while one was conducted on knee arthroplasty [30]. Regarding the disease phase, six studies and one study focused on the acute and/or subacute phase, respectively [9, 13, 18, 22, 31, 34], and the chronic phase [36] of stroke, while one study targeted rural veteran stroke survivors [16]. The last three studies (one on knee arthroplasty and two on stroke) did not specify the disease phase.

The results of the study population are presented in Table 1 and Appendix 5.

**Table 1:** Study characteristics based on country, type of illness, objective and study population

Author	Number of studies	Country	Illness	Objective	Patient characteristics
**Wagner et al., 2011**	1	USA	Patients with moderate to severe motor impairment of the upper limbs, caused by stroke, admitted to a center 6-months after stroke	Determine the additional cost of robot-assisted therapy and tested its cost-effectiveness	Male, n (%): RT: 47 (96) ICT:48 (96) UC: 27 (96) Female, n (%): RT: 2 (4) ICT: 2 (4) UC: 1 (4) **Total: 127** Age, Mean (SD) RT: 66(11) ICT:64 (11) UC: 63 (12)
**Hesse et al.,2014**	2	Germany	Supratentorial stroke patients (hemorrhagic or ischemic)	Evaluate the cost-effectiveness and the effectiveness of RAGT versus IAT to restore the motor function in the moderately to severely affected patient after stroke	Male, n (%): RAGT+IAT: 13 (52) IAT: 15 (60) Female, n (%) RAGT+IAT: 12 (48) IAT: 10 (40) **Total: 50** Age, Mean (SD) RAGT+IAT: 71.4 (15.5) IAT: 69.7 (16.6)
**Stefano et al., 2014**	3	USA	Patients with stroke-induced upper limb impairment, acute or subacute hemiparetics. Shoulder, elbow or wrist muscle groups (patients with acute (< or =1 week of onset), unilateral, ischemic embolic, or thrombotic stroke)	To compare the costs of such therapy to the control therapies used in the NeReBot clinical trials run thus far	Male, n (%): RT: 9 (64.29) UC: 6 (75) Female, n (%) RT: 5 (35.71) UC: 2 (25) **Total:22** Age, Mean (SD) RT: 72.4 (7.1) UC: 75.5 (4.8)
**Lloréns et al., 2016**	4	Spain	Chronic Outpatient with stroke with residual hemiparesis	- To evaluate the clinical effectiveness of a VRT program in the balance recovery of individuals with hemiparesis after stroke in comparison with an in-clinic program. - To compare the subjective experiences - To contrast the costs of both programs.	Male, n (%): VRT: 10 (66.7) Control: 7 (46.7) Female, n (%) VRT: 5 (33.3) Control: 8 (53.3) **Total:30** Age, Mean (SD) VRT: 55.47(9.63) Control: 55.60(7.29)
**Bustamante Valles et al., 2016**	5	Mexico	Hemiplegic patients in chronic phase secondary to stroke	To develop and deliver an effective and cost and labor cost-effectiveCost-effective method of poststroke rehabilitation that encouraged continued rehabilitation and was more or as effective as traditional physical and occupational therapy approaches	Male, n (%): RG: 3 (30) Control:4 (40) Female, n (%) RG: 7 (70) Control: 6 (60) **Total:20** Age, Mean (SD) RG: 44.1(12.53) Control: 64.1(8.38)
**Housley et al., 2016**	6	USA	Stroke survivors with unilateral ischemic or hemorrhagic stroke within the previous 24 months	To examine the efficacy of using a home-based, tele robotic-assisted device to: improve functional ability, reduce depression symptoms, and create a satisfactory experience, increase access to, and monitor participant utilization of cost-effective rehabilitation compared to the cost of clinic-based therapy for rural stroke survivors	Male, n (%):19 (95) Female, n (%):5 (5) **Total: 20** Age, Mean (SD) VRT: 55.47(9.63) Control: 55.60(7.29)
**Adie et al., 2017**	7	UK	Patients with weakness following a stroke with the previous six months	Evaluate the efficacy of using the Wii^TM^ to improve affected arm function after stroke	Male, n (%): Wii^TM^: 66 (56.41) CT: 65 (53.39) Female, n (%) Wii^TM^: 51 (43.59) CT: 53 (46.61) **Total: 235** Age, Mean (SD): Wii^TM^: 73.1(5.8) Usual care: 73.0(5.5)
**Islam and Brunner, 2019**	8	Denmark, Norway, and Belgium	Patients with unilateral ischemic or hemorrhagic stroke within the 3 first months after suffering a stroke	To explore cost considerations of VR training in stroke	Male, n (%): VRT: 42(68) C6T: 35 (60) Female, n (%) VRT: 20 (32) CT: 23 (40) **Total=120** Age, Mean (minimum-maximum) VRT: 62 (23–89) CT: 62 (41–87)
**Prvu Bettger et al., 2020**	9	USA	Patients with TKA	To examine costs and clinical noninferiority of a VPT program compared with traditional PT care after TKA	Male, n (%): VPT: 61(40.40), UC: 53 (34.60) Female, n (%): VPT: 90 (59.60) UC: 100 (65.40) **Total:304** Age, Mean (SD) VPT: 65.40 (7.7) Control: 65.1 (9.2)
**Rémy-Néris et al., 2021**	10	France	Patients aged 18 to 80 years, 3 weeks to 3 months poststroke with a FM Assessment score of 10 to 40 points	To determine differences in cost utility between the two interventions at D30 and M12	**Male, n (%):** Exo group: 73(67.59) Control:67(62.62) **Female, n (%):** Exo group: 35(32.42) Control:40(37.38) **Total:215** **Age, Mean (SD):** Exo group: 73(67.59) Control:67(62.62)
**Fernandez-Garcia et al., 2021**	11	UK	Patients with moderate or severe upper limber functional limitation from first-ever stroke	To determine whether RAT is cost-effective compared with an EULT programme or usual care	Male, n (%): RAT: 156 (61%) EULT:159 (61%) Control: 153 (60%) Female, n (%): RAT: 101 (39%) EULT: 100 (39%) UC: 101 (40%) **Total:770** **Age, Mean (SD):** RAT: 59.9 (13.5) EULT: 59.4 (14.3) UC: 62.5 (12.5)

*D30* 30th day, *EULT* Enhanced Upper Limb Therapy, *FM* Fugl Meyer, *IAT* Individual Arm Therapy, *ICT* Intensive Comparison Therapy, *M12* Twelfth month, *NeReBot* Neuro-Rehabilitation-Robot, *PE* Physical Exercise, *PT* Physical Therapy, *RAGT* Robot-Assisted Gait Therapy, *RAT* Robot-Assisted Therapy, *RT* Robot Therapy, *SD* Standard Deviation, *TKA* Knee Arthroplasty, *UC* Usual Care, *UK* United Kingdom, *USA* United States of America, *USD* United States Dollars, *VA* Veterans Affairs, *VPT* Virtual Physical Therapy, *VRT* Virtual Realty-based Telehabilitation, *Wii*^*TM*^ Wii Sports

### Foundational choices of economic evaluation

All studies evaluated the cost-effectiveness of the intervention compared to usual care. The majority of studies (7/11) adopted a healthcare system perspective for cost evaluation. The time horizons varied, ranging from a few weeks to 12 months, but were short for most studies (6/11 < 6 months). Four studies conducted CUA, while the remaining seven performed CMA. Two studies discounted costs and health outcomes.

Studies included in this systematic review analyzed the cost-effectiveness of motor rehabilitation interventions involving the use of digital technologies, comparing them to usual care through randomized controlled trials.

The majority of studies mentioning an assessment perspective adopted a healthcare system perspective for cost evaluation, except for two studies that adopted a societal perspective [18, 36]. It was not possible to clearly identify the assessment perspective of some studies [13, 22, 34].

The time horizon varies from four weeks [18], and extrapolation beyond one year was conducted in one study [9] to estimate the long-term cost-effectiveness of the intervention. Five studies adopted a time horizon of 6 months or more, while six studies adopted a time horizon of less than 6 months [1, 9, 30, 31, 36]. Of the eleven studies included in this review, four CUA used a two-dimensional health outcome measure to assess health-related quality of life. These studies employed generic quality of life assessment questionnaires such as the EuroQol 5-Dimensions (EQ-5D) [9, 31] or the Health Utilities Index (HUI) [36]. The remaining seven studies were CMAs that used a unidimensional health outcome measure, assessing physical outcomes such as patient capacity improvement using tools such as the Action Research Arm Test (ARAT) or the Fugl-Meyer Assessment (FMA). Importantly, no cases of CEA were included in this review.

The types of costs identified in the studies varied depending on the chosen evaluation perspective. However, these costs generally included equipment costs, healthcare professionals' costs, medication costs, home visit costs, administrative and overhead costs, and social care costs. The majority of studies estimated these costs based on single-site evaluation. Some studies assessed costs across multiple sites [18, 19, 31], while one study generalized cost estimates nationwide [9]. The specific types of costs identified in the various studies are detailed in Appendix 6. The uncertainties surrounding the cost-effectiveness results were assessed in three studies [1, 9, 36], while discounting of the cost and effectiveness results was carried out in two studies [9], [36]. The results of the foundational choices of economic evaluation are presented in Table 2*.*

**Table 2:** Results of the foundational choices of economic evaluation

Author	Type of economic evaluation	Time horizon	Perspective	Type of cost	Main outcome measure	Discounting
**Wagner et al., 2011**	CUA based on HUI	36 Weeks	Societal		ICER	YES
**Hesse et al., 2014**	CMA	3 months	Unspecified		Cost saving	NO
**Stefano et al., 2014**	CMA	3 months	Italian National Health system		Cost saving	NO
**Lloréns et al., 2016**	CMA	12 weeks	Unspecified		Cost saving	NO
**Bustamante Valles et al., 2016**	CMA	8 weeks	Unspecified		Cost saving	NO
**Housley et al., 2016**	CMA	3 months	VA health system		Cost saving	NO
**Adie et al., 2017**	CUA based on EQ-5D	6 months	Heath and social service		QALY and healthcare costs	NO
**Islam and Brunner, 2019**	CMA	4 weeks	Societal perspective		Cost saving	NO
**Prvu Bettger et al., 2020**	CMA	12 Weeks	Health care system		Cost saving	NO
**Rémy-Néris et al., 2021**	CUA based on EQ-5D	12 months	Health care system		ICER	NO
**Fernandez-Garcia et al., 2021**	CUA based on EQ-5D	6 months	UK-NHS		ICER	YES

*CMA* Cost-minimization analysis, *CUA* Cost utility analysis, *EQ-5D* EuroQol-5 Dimensions, *HUI* Health Utilities Index

### Quality assessment

The quality scores of the included studies ranged from 52% (acceptable quality) to 100% (very good quality), with generally higher scores for CUAs than for CMAs.

**Fig. 2:**
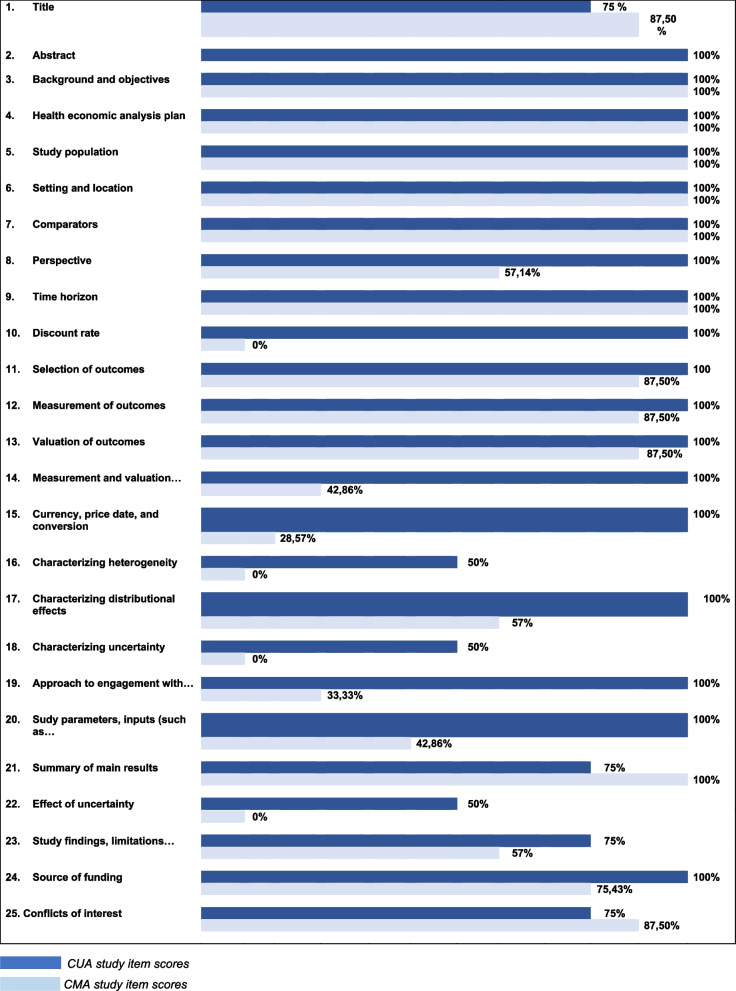
Quality assessment of the included studies

On the basis of the CHEERS checklist, the articles included in the review had quality scores ranging from 52 [18] to 100 [9] out of a maximum score of 100. All quality scores are presented in Appendix 7. Trials with a time horizon of 12 months or less received 'NA' for the items 'discount rate', 'patient and public involvement', 'model justification and description', and 'analysis and assumptions'.' Two studies [9, 36] performed cost and consequence discounting, considering the five-year amortization period used in these studies. Four trials with a quality score exceeding 80 out of 100 were classified as high-quality economic evaluations, while all other trials with scores ranging from 40 to 70 out of 100 were classified as medium-quality economic evaluations (see Appendix 7). We present the quality scores obtained by the CUA and CMA for each item on Fig. 2.

Notably, the previously mentioned inapplicable items for all studies included are not presented in the graph, thereby reducing the number of items presented to 25 instead of 28 in the CHEERS checklist.

### Evidence for the cost-effectiveness of motor rehabilitation assisted by digital technology interventions

This section summarizes the cost-effectiveness of rehabilitation interventions. In summary, out of the 11 studies included, the intervention evaluated in two of them was deemed not cost-effective, that evaluated in 2 studies was considered to have cost-effectiveness equivalent to standard care, and that evaluated in seven studies was deemed cost-effective.

The health outcomes were similar between the intervention and usual care groups in the 7 CMA studies. Additionally, the average QALYs between the intervention group and the control group did not significantly differ among the 4 CUA studies (see Table 3).

**Table 3:** Health outcomes (QALYs) of CUA included

Autho**r**	Time horizon for calculating the average cost	QALY-mean (CI)	Mean difference	*p* value	Conclusion
**Wagner et al., 2011**	36 weeks	Robot* (*n*=49): QALY= 0.37 (0.03) Intensive comparison therapy (*n*=50): QALY= 0.36 (0.03) Usual care: (*n*=28): QALY= 0.30 (0.05)	0.05 ±0.07	-	No difference in mean quality-adjusted life years between the two groups
**Adie et al., 2017**	6 months	Wii^TM^ (*n*=85): QALY=0.26±0.08 Arm exercises(*n*=89): QALY=0.26±0.08	There was no difference in mean QALY between the two groups	0.86	No difference in mean quality-adjusted life years between the two groups
**Rémy-Néris et al., 2021**	**12 months**	Control (*n*=108): QALY=0.47±0.26 Exo group* (*n*=107): 0.48±0.25	There was no difference in mean QALY between the two groups-	*P*=0.87	No difference in mean quality-adjusted life years between the two groups
**Fernandez-Garcia et al., 2021**	12 months **(after extrapolation)**	RAT* (*n*=257): QALY= 0.21±0.12 EULT (*n*=259): QALY= 0.23±0.10 Usual care: (*n*=259): QALY= 0.21±0.11±	0.00 (−0.20 to 0.20)	0.995	No difference in mean quality-adjusted life years between the RAT group and control group

Five studies, including four CMA studies and one CMA study [30], prospectively collected the resources consumed per patient and per intervention over the entire study period for evaluation. Among these studies, Prvu Bettger et al. [30] concluded that the cost of the intervention was lower than that of its control. Other studies reported either cost equivalence between the intervention and its control [1, 31, 36] or a higher cost of the intervention compared to the control [9]. The cost evaluation of interventions in six other studies was based on models incorporating assumptions about various cost components, such as the number of personnel involved in rehabilitation, the duration and frequency of rehabilitation sessions, and the lifespan of equipment. Among these studies, one reported the effectiveness of usual care, while four reported the effectiveness of the intervention itself.

The cost outcomes are presented in the table below (Table 4):

**Table 4:**
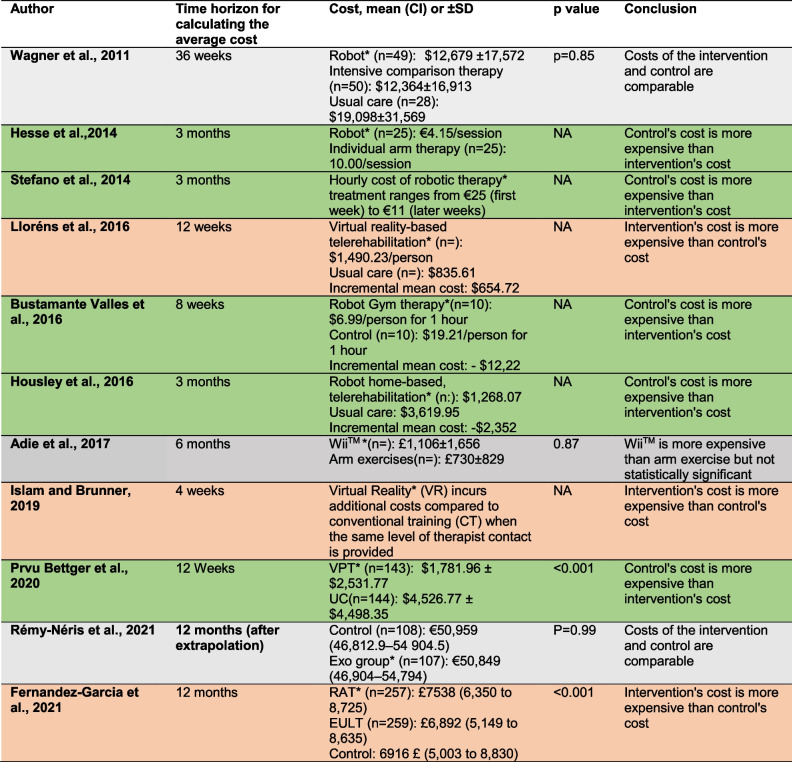
Cost outcomes of included studies

* Indicates the intervention assessed in the study

The cost of intervention is greater than that of usual care

The cost of intervention is lower than that of usual care

Intervention and usual care costs are both comparable

The general criteria considered for cost analysis are presented in the table below. Since the evaluation perspective was not provided in all studies, it was not included in our cost analysis criteria (Table 5).

**Table 5:** Conclusions of the cost outcomes of the included studies

Author	Rehabilitation technology (model)	HT≥6 mois	Identification, measurement, and valuation of resources in both arms	Conclusion
**Wagner et al., 2011 **	Robot	YES	YES	Lower cost
**Hesse et al.,2014**	Robot (Bi-Manu Track)	NO	NO	Lower cost
**Stefano et al., 2014 **	Robot (NeReBot)	NO	NO	Lower cost
**Lloréns et al., 2016 **	Virtual reality	NO	NO	Higher cost
**Bustamante Valles et al., 2016**	Robot (Ness for upper extremity, the Motomed Viva 2 for upper extremities)	NO	NO	Lower cost
**Housley et al., 2016 **	Robot	NO	NO	Lower cost
**Adie et al., 2017 **	**Wii**^**TM**^** sport**	YES	YES	Equivalent
**Islam and Brunner, 2019**	Virtual reality (Bi-Manu-Trainer (Reha-Stim Medtech Ltd., Switzerland))	NO	YES	Higher cost
**Prvu Bettger et al., 2020**	Virtual reality	YES	YES	Lower cost
**Rémy-Néris et al., 2021**	**Robot (Armeo Spring® HOCOMA)**	YES	YES	Equivalent
**Fernandez-Garcia et al., 2021**	Robot (MIT-Manus robotic)	YES	YES	Higher cost

HT≥6: When the temporal horizon of a study exceeds 6 months, the answer is YES; otherwise, it is NO

**Fig. 3:**
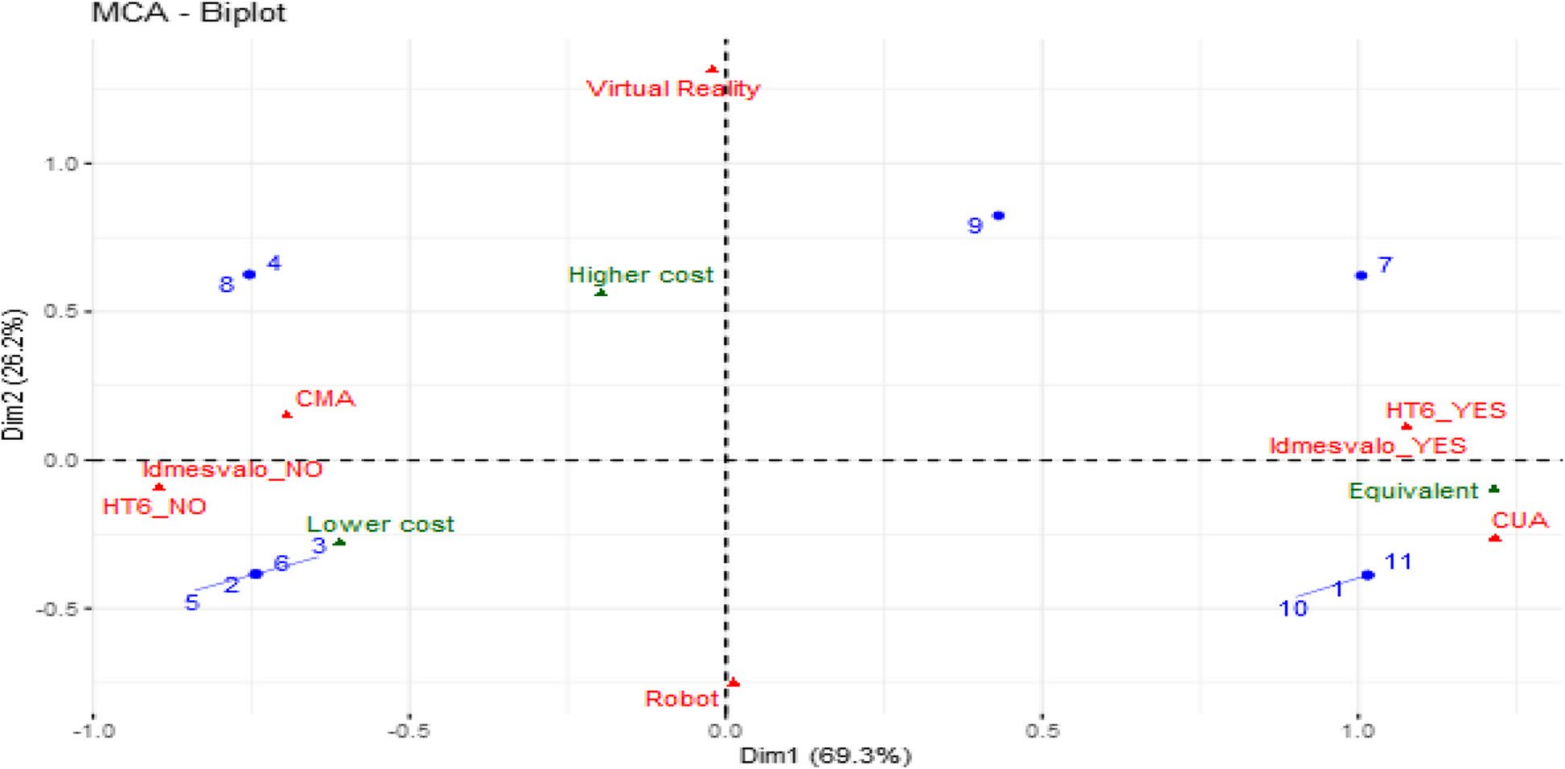
Multiple correspondence analysis of determinants of cost outcomes. HT6_YES: when the time horizon is equal to or greater than 6 months. HT6-NO: when the time horizon is lower than 6 months. CMA: Cost Minimization Analysis . CUA: Cost Utility Analysis. Higher cost: The cost of intervention higher than that of usual care. Lower cost: The cost of intervention is lower than that of usual care. Equivalent: The cost of intervention is the same as the comparator's. Idemesvalo_YES: Applies to studies that prospectively collect resources used in each group over the entire time horizon, following economic analysis guidelines. 1, 2, 3..., 11: Represents the number of studies

To examine the association between the increase in intervention cost compared to that of his comparator and other explanatory variables, such as the time horizon (greater than, equal to, or less than 6 months), the methodological quality of the economic evaluation, the type of technology assessed (virtual reality or robotics), and the type of economic analysis method conducted (CMA or CUA), we conducted a multiple correspondence analysis (MCA), the results of which are presented in the figure below (Fig. 3):

The results indicate that studies utilizing a CUA to evaluate an intervention, prospectively collecting resources and assessing them in accordance with healthcare program economic evaluation standards, over a 6-month or longer time horizon, are more likely to yield similar cost outcomes compared to the comparator. Conversely, studies with an observation period of less than 6 months and not adhering to resource collection and evaluation recommendations tend to demonstrate a lower intervention cost than the comparator. The results CMA does not show a clear trend regarding the cost-effectiveness of different digital technologies. However, interventions based on virtual reality appear more likely to incur higher costs compared to conventional care.

The conclusion regarding the cost-effectiveness of the intervention compared to its comparator was based on estimating the ICER in three out of the four CUA patients. Among these, one demonstrated the effectiveness of the intervention, another concluded the inefficiency of the intervention, while the last indicated equivalence between the intervention and its comparator in terms of cost-effectiveness. The fourth CUA [1] estimated the average QALYs and costs of the compared interventions but did not provide incremental costs, QALYs, or the ICER. However, there was equivalence between the intervention and its comparator in terms of cost-effectiveness. On the other hand, the seven CMAs conducted indirect comparisons based on the costs and effectiveness of the intervention and its comparator without calculating ICERs. Among these studies, one reported the cost-effectiveness of usual care, while the other six reported the cost-effectiveness of the intervention compared to its comparator.

Three out of the eleven included studies, specifically two out of the four CUA studies and one out of the seven CMA studies, conducted sensitivity analyses. These analyses did not alter the final conclusions on cost-effectiveness (Table 6).

**Table 6:** Study characteristics based on decision rules, ICER, conclusion of the study and sensibility analysis

Author	Decision rules	ICER	Conclusion	Sensibility analysis
**Wagner et al., 2011**	Unspecified	-25,770 suggesting dominance of the robot over user care	Robot is cost-effective	The results were not sensitive to the source of veteran’s affairs (VA) cost data. Changing of the discount rate had a very little effect on the results because the robot’s lifespan was relatively short
**Hesse et al.,2014**	Unspecified	Not calculated	The treatment costs for Robot-assisted gait therapy were less than individual arm therapy’s	Note done
**Stefano et al., 2014**	Unspecified	Not calculated	Robotic technology generates savings cost for the National Health Care System.	Not done
**Lloréns et al., 2016**	Unspecified	Not calculated	VR-based telerehabilitation programs can save costs, mainly derived from transportation services	Not done
**Bustamante Valles et al., 2016**	Unspecified	Not calculated	Robot Gym therapy is more or as cost-effective as traditional therapy	Not done
**Housley et al., 2016**	Unspecified	Not calculated	The results of this study demonstrate a substantial (64.97%) savings for the VA healthcare system.	Not done
**Adie et al., 2017**	Not done	Wii^TM^ arm is dominated by the usual care	The Wii^TM^ was more expensive than arm exercises The estimate of probability that the Wii^TM^ arm is dominated is 0.866	The use of simple imputation did not change the observed findings
**Islam and Brunner, 2019**	Unspecified	Not calculated	No cost-savings for VR were achieved due the same therapist time spent in VR and CT Cost saving increase when more patients train with VR	Not done
**Prvu Bettger et al., 2020**	Unspecified	Not calculated	Patients who received VPT had lower total posthospital costs at 12 weeks compared with patients who received usual care. Outcomes: All outcomes for all time-periods assessed were similar between groups	Not done
**Rémy-Néris et al., 2021**	WTP values: €50-100,000/QALY (59,240-118,480$US)	Robot is not cost-effective	There was no between-group difference in cost utility at M12 (12 months)	Not done
**Fernandez-Garcia et al., 2021**	WTP values: £0, £10 000, £20 000, £30 000, £50 000	£6 095	The usual care was the most cost-effective rehabilitation method for patients who had a stroke with functional limitation of the upper limb	The sensitivity analysis confirmed the results of the base-case analysis. The different scenarios explored did not change the direction of the results. Robot-assisted training was consistently dominated on average by usual care, regardless of the scenario. The variations in costs imputed to missing data did not alter the conclusions. Extending the lifespan of the robotic gym system also did not affect the cost-effectiveness of the interventions.

*CT* clinical trial, *ICER* incremental cost-effectiveness ratio, *QALY* quality adjusted life years, *VPT* virtual physical therapy, *VR* virtual reality, *Wii*^*TM*^ Wii Sports ^TM^, *WTP* willingness to pay

#### Dominance ranking framework

Based on the dominance ranking framework, interventions evaluated in four studies were considered not cost-effective in motor rehabilitation, while those assessed in six studies were deemed cost-effective. It is not possible to determine the cost-effectiveness of the intervention evaluated in the French study without considering the willingness to pay for 1 QALY (Table 7).

**Table 7:** Dominance ranking matrix of the included studies

Cost	Health benefit	Implication for decision makers	Total number^N° of study^
**+**	**-**	**Reject intervention**	**None**
**0**	**_**	**Reject intervention**	**None**
**+**	**0**	**Reject intervention**	**2 **^**(4), (11)**^
**-**	**-**	**No obvious decision: judgment required on whether intervention is preferable considering cost-effectiveness measures and priorities/willingness to pay**	**None**
**0**	**0**	**No obvious decision: judgment required on whether intervention is preferable considering cost-effectiveness measures and priorities/willingness to pay**	**2 **^**(7), (10)**^
**+**	**+**	**No obvious decision: judgment required on whether intervention is preferable considering cost-effectiveness measures and priorities/willingness to pay**	**None**
**-**	**0**	**Favor intervention: accept intervention**	**7 **^**(1), (2), (3), (5), (6), (8), (9)**^
**0**	**+**	**Favor intervention: accept intervention**	**None**
**-**	**+**	**Favor intervention: accept intervention**	**None**

*+ =* higher cost/better health outcome; 0 = equal cost/equal health outcome; – = lower cost/poorer health outcome

**Fig. 4:**
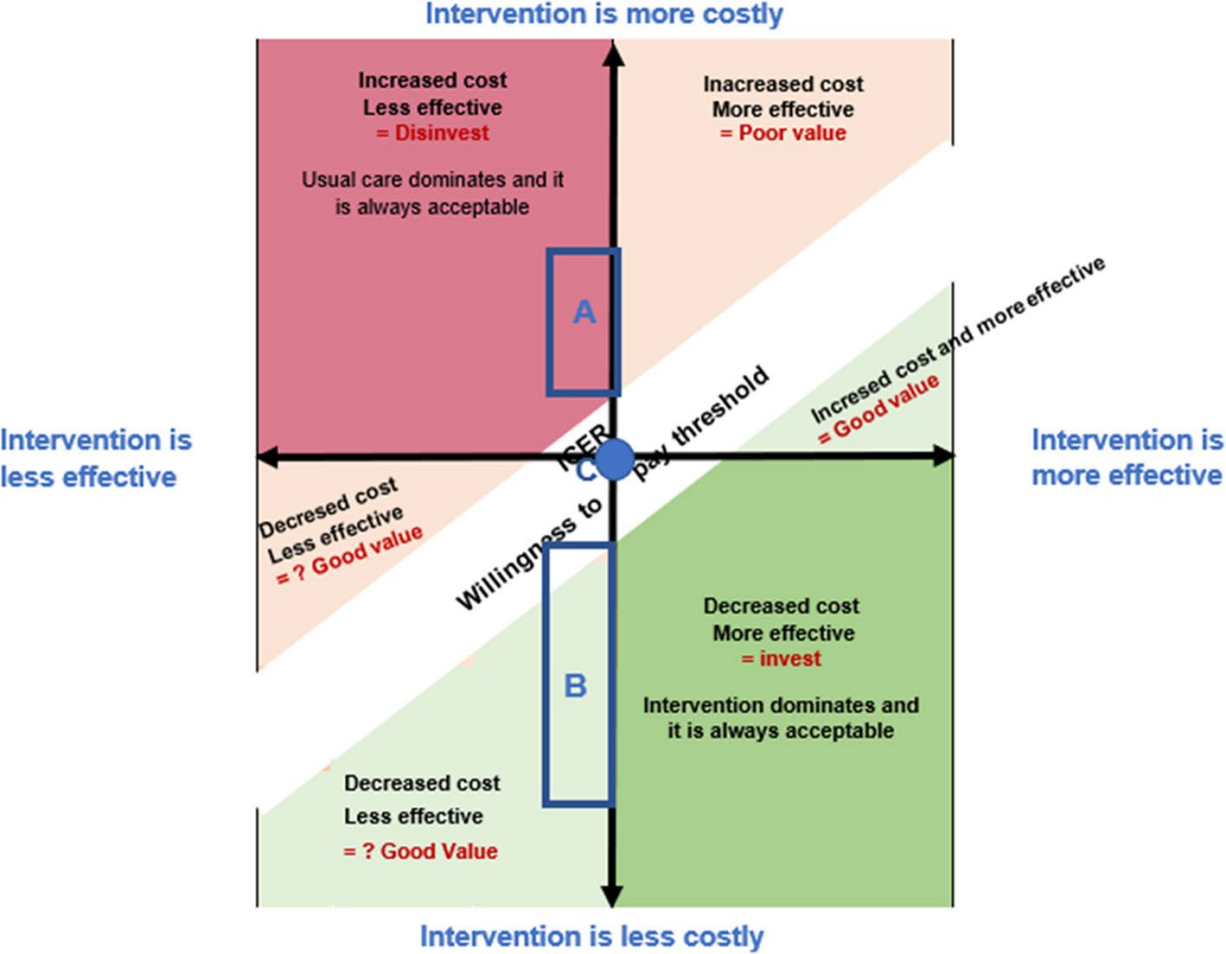
Cost-effectiveness plane of included studies. **A** Two studies are located in this area: Fernandez-Garcia et al. and Lloréns et al. **B** Seven studies are located in this area: Wagner et al., Hesse et al., Stefano et al., Bustamante Valles et al., Housley et al., Adie et al., and Islam and Brunner. **C** Two studies are located in this area: Rémy-Néris et al. and Prvu Bettger et al.

We complemented our study by positioning each included study on a cost-effectiveness plane based on the results obtained in the previous paragraph (see Fig. 4*)*.

The cost-effectiveness outcomes corroborate the previous conclusions of the cost analysis.

## Discussion

The objective of this study was to assess the available evidence on the cost-effectiveness of digital technologies for the motor rehabilitation of individuals with neurological disorders. Our results support that there is significant heterogeneity among studies in terms of design, methods, health outcomes, and cost-effectiveness outcomes. Eleven studies, including four CUA studies [1, 9, 31, 36] and seven CMA studies [4, 13, 16, 18, 22, 30, 34], were included in this review. The vast majority of the included studies focused on poststroke rehabilitation [1, 4, 9, 13, 16, 18, 22, 31, 34, 36].

Among the eleven studies examined, seven [4, 13, 16, 18, 30, 36] demonstrated the cost-effectiveness of the intervention compared to conventional care, while two [1, 31] showed equivalence in terms of cost-effectiveness between the intervention and its comparator. However, two others indicated the cost-effectiveness of the comparator [9, 22]. It is worth noting that among the seven studies favoring the intervention, only one was a CUA, with the others being a CMA. Additionally, half of the CUAs, representing two studies out of four, showed equivalence in terms of cost-effectiveness between the intervention and its comparator. Finally, one CUA, accounting for 25% of the total CUA, revealed that conventional care was more economically effective than the intervention. The cost of the device influences the cost of robotic technology, whereas for rehabilitation based on virtual reality or video games, the cost of the staff is most important.

Six out of seven studies using a CMA concluded that the intervention was cost-effective. In contrast, only one study out of the four based on a CUA concluded that the intervention was cost-effective. As illustrated in the graph of the multiple correspondence analysis, it appears that the temporal horizon has an impact on the effectiveness of motor rehabilitation based on the use of digital technologies. Indeed, among the four studies [1, 9, 18, 31] concluding inefficiency or equivalence of the intervention, three [1, 9, 31] adopted a temporal horizon of six months or longer. In contrast, only one study [36] adopted a temporal horizon of six months or more among the seven studies that reported the cost-effectiveness of the intervention. The nature of the evaluated interventions could explain these results. For instance, in interventions involving medications, the usual initial treatment involves their administration at specific times, followed by a monitoring period, leading to decreasing costs over a time horizon, thus justifying the use of the gamma law in the economic evaluation of health interventions. For rehabilitation interventions, costs are more likely to remain constant over the time horizon, notably due to device amortization over a long lifespan (often 5 years), resulting in a constant average device cost over the entire time horizon. While certain costs such as personnel, medication, and administrative costs may decrease over time, their impact on total average costs would still be limited given the high acquisition price of certain rehabilitation devices. Finally, in rehabilitation, interventions are more effective when they are early and intensive. Thus, an extended time horizon does not guarantee better outcomes, either in terms of cost or health outcomes, as it could reflect the chronicity of the pathology.

Two studies [18, 36] adopted a societal perspective,one concluded the cost-effectiveness [36] of the intervention, while the other reached the opposite conclusion [18]. Fivestudies [1, 9, 13, 30, 31] have adopted a health system perspective. We did not find any indication regarding the perspective of economic evaluation in the other studies.

Ten [1, 4, 9, 13, 16, 18, 22, 31, 36] out of eleven studies focused on stroke. Two studies [4, 22] included only patients in the chronic phase of stroke, and both reported the cost-effectiveness of the intervention for patients with motor disabilities. Eight studies thus included stroke patients in the acute and/or subacute phase, as well as in the chronic phase [1, 9, 13, 16, 18, 31, 34, 36]. Four studies [13, 16, 34, 36] concluded that the intervention was cost-effective for individuals with motor disabilities. Due to the heterogeneity among the included populations associated with a variety of interventions, study perspectives, and temporal horizons, among other factors, these results do not allow us to conclude whether there is an association between the disease phase and cost-effectiveness.

Using the CHEERS checklist to assess the quality of the trials included in this systematic review, we found that seven studies [4, 13, 16, 18, 22, 30, 34] were of moderate quality, while four [1, 9, 31, 36] were of high quality. The quality scores of economic studies vary according to the analysis method used. Studies applying CUA obtained the highest scores, ranging from 80 to 100%, according to the CHEERS checklist. In contrast, studies based on a CMA present more modest quality scores, ranging between 52 and 73%. These results reflect the recommendations of the French National Authority for Health (HAS) [12] in favor of CUA or CEA in the economic evaluation of health strategies. When these methods are rigorously applied, they generate an ICER on which decision-makers can rely to adopt care programs and allocate health resources.

Similarly, in our review, the reviews by Lo [24] and Kairy [20] also revealed that the majority of the included studies reported the cost-effectiveness of the intervention. Our review has a sample size of 1825, whereas Lo's has a sample size of 213. Although our study incorporates diverse populations and various rehabilitation technologies, the sample size ensures sufficiently robust conclusions.

Regarding the assessment of the quality of the included studies, our findings are similar to those found in the review by Lo et al. Indeed, 20% of their included studies were of high quality, compared to 36.36% in our review. The differences found could be explained by the fact that we included more recent studies, with 91% of the included studies being conducted in the last decade. Additionally, unlike other studies, we included only RCTs. Finally, we included more CUAs in our review.

This review has several strengths. First, it provides the most recent data on the cost-effectiveness of rehabilitation interventions using digital technologies. Indeed, ten out of eleven included studies were published in the last ten years, with four of them published in the last four years. These studies are thus based on updated methodological guidelines for economic evaluations [5, 8, 12, 14, 17, 25].

We opted for a systematic review due to its ability to comprehensively and rigorously synthesize the cost-effectiveness evidence of digital technologies in motor rehabilitation. This methodology offers several advantages over other methods. It produces results that are more representative of the target population than those obtained from a single clinical trial or theoretical model, which are limited to a specific context. By integrating multiple studies, it enhances the reliability of conclusions and allows for better reproducibility of the analysis. The use of the latest version of the CHEERS checklist enabled a standardized and rigorous evaluation of the methodological quality of the included studies. The systematic process of searching, selecting, and analyzing studies based on the PRISMA guidelines ensures a robust methodological approach. By synthesizing multiple data sources, this method allows for more nuanced and relevant conclusions to guide decision-making regarding the adoption of digital technologies in motor rehabilitation. It thus provides a solid basis for evaluating the cost-effectiveness of motor rehabilitation interventions assisted by digital technologies.

The results of this systematic review highlight several potential benefits of using digital technologies for motor rehabilitation. Firstly, these interventions seem to enable more effective rehabilitation through the possibility of repetitive, intensive exercises tailored to the specific needs of patients. Secondly, by improving functional and motor abilities, they could have a positive impact on patients' health-related quality of life. Thirdly, several studies included in the review concluded that these interventions were cost-effective compared to conventional approaches, thereby allowing for cost savings.

However, these technologies should not replace but rather complement conventional rehabilitation methods to address current sector challenges such as staff shortages and limited access. Their integration would allow for an increase in the number of rehabilitation sessions accessible to patients, offering the possibility of performing certain exercises at home or in other locations besides specialized centers. This would free up space in the centers for patients with greater needs for supervision and support. Ultimately, this could prevent interruptions or lack of care for some patients, thereby improving the overall provision and accessibility of rehabilitation services.

The sample size of our review is one of its strengths, particularly in the context of RCTs. This ensures adequate statistical power and robust estimates.

This systematic review, although rigorous in its methodology, presents certain limitations that should be considered when interpreting the results. One of the main limitations of this systematic review is its restriction to randomized clinical trials. Although this approach ensures high methodological rigor, it considerably reduced the number of eligible studies. Indeed, other potentially relevant economic studies based on models such as Markov models were excluded. As a result, the vast majority of the included studies (10/11) focused on strokes, limiting the generalization of the results to other neurological conditions.

Another major limitation is that this review did not consider gray literature, focusing only on published studies. However, the inclusion of unpublished gray literature could have provided additional interesting data.

Finally, an important limitation is the absence of subgroup analyses. This is explained by the significant heterogeneity observed between the included studies, particularly in terms of the studied populations, the types of interventions compared, the economic evaluation methods used (CMA and CUA), and the structural choices of the economic evaluation (time horizon, perspective, discount rate, sensitivity analyses).

To address these limitations, future research should consider including other types of economic studies in addition to randomized trials, such as analytical decision models that can capture long-term outcomes. The inclusion of unpublished gray literature would provide a more comprehensive view of the existing evidence. We also recommend conducting a meta-analysis as done in a recent study [33], if the studies allow it, when model-based studies are included to provide an overall estimate that will more easily answer the question of the cost-effectiveness of these interventions.

Finally, it will be relevant to assess the budgetary impact of integrating these technologies into health systems.

## Conclusion

This systematic review provided the latest data on the cost-effectiveness of digital rehabilitation interventions for neurological disorders. Out of the 11 studies included, 7 concluded the cost-effectiveness of these interventions for the target population. Associations were observed between the economic evaluation method used (cost-utility analysis vs. cost-minimization) and cost-effectiveness outcomes, as well as between time horizon and cost-effectiveness.

These findings suggest that integrating rehabilitation technologies should complement rather than replace conventional approaches to more effectively achieve medical and economic goals. Adoption could alleviate healthcare professionals' and patients' workload by reducing physical efforts and travel through tele-rehabilitation. Further studies, including decision modeling, are needed to better understand the long-term outcomes of these interventions. Evaluating their financial impact on healthcare systems would also be relevant for facilitating their integration.

### Dictionary of economic terms

#### Cost minimization analysis (CMA)

CMA is an economic evaluation method used to compare different treatment or management options, focusing solely on their respective costs. In the context of this review, the studies using this approach compared the cost of implementing the digital technology-based intervention to the cost of implementing conventional motor rehabilitation care (traditional physiotherapy).

#### Cost-utility analysis (CUA)

CUA is an economic evaluation method used to compare different interventions by considering both their respective costs and their health benefits, expressed in terms of QALYs. In the context of this review, the studies using this approach compared the cost of implementing the digital technology-based intervention to the cost of implementing conventional motor rehabilitation care. The results of these studies are therefore presented as the relative costs to obtain an additional QALY unit with the intervention compared to conventional care.

#### Discount rate

In economic evaluations, a discount rate is applied to bring future estimated costs and benefits to their present value. This is typically done when the time horizon is beyond one year. This practice is based on the principle that an amount available immediately is more valuable than the same amount received in the future, due to present-time preference and the opportunity cost of money.

#### Incremental Cost-Effectiveness Ratio (ICER)

The ICER is an indicator that reflects the additional cost to be incurred to obtain an additional unit of health outcome with the new intervention compared to the old one. It helps decision-makers assess whether the additional benefits of the new intervention are worth the additional costs compared to the existing care or standard practices.

#### Perspective

The perspective refers to the viewpoint adopted for the evaluation of costs. The healthcare system perspective considers all costs related to healthcare (for the patient, health insurance, mutual insurance companies, etc.), except for costs related to patients' loss of productivity. The societal perspective, on the other hand, differs from the healthcare system perspective by incorporating the costs of lost productivity, which are not considered in the healthcare system perspective.

#### QALY

Quality-adjusted life years are calculated from generic measures of health-related quality of life, such as the EQ-5D, SF-36, or HUI. The scores obtained from these questionnaires are converted into utility scores, which are then multiplied by the duration during which a participant lived with that utility.

#### Sensitivity analysis

Allows assessing the robustness of the cost-effectiveness analysis results in the face of uncertainties surrounding the estimation of costs and health outcomes.

#### Time horizon

Refers to the duration over which costs and outcomes of an intervention are assessed

### Supplementary Information

Supplementary Material 1.

## Data Availability

No datasets were generated or analysed during the current study.

## Footnotes

1 Physical disabilities such as vision problems, lack of energy, and motor deficiencies hinder walking training. Additionally, lack of motivation to engage in any type of exercise, alterations in patients' mental health status, and fear of falling and losing balance are also obstacles

2 The constraints related to managing schedules and family responsibilities are social barriers that hinder physical rehabilitation

3 The lack of family and social support, transportation and accessibility challenges, as well as a lack of awareness of opportunities, are obstacles to physical rehabilitation

4 The shortage of access to programs, lack of tailored interventions, and absence of exercise options leading to boring or monotonous training sessions

## References

[1] Adie K, Schofield C, Berrow M, Wingham J, Humfryes J, Pritchard C, James M, Allison R (2017). Does the use of Nintendo Wii SportsTM improve arm function? Trial of WiiTM in Stroke: a randomized controlled trial and economics analysis. Clin Rehabil.

[2] Bendixen RM, Levy CE, Olive ES, Kobb RF, Mann WC (2009). Cost effectiveness of a telerehabilitation program to support chronically ill and disabled elders in their homes. Telemed J E Health.

[3] Brusco N, Voogt A, Nott M, Callaway L, Mansoubi M, Layton N (2022). Meeting Unmet Needs for Stroke Rehabilitation in Rural Public Health: Explorative Economic Evaluation of Upper Limb Robotics-Based Technologies through a Capabilities Lens. Societies.

[4] Bustamante Valles K, Montes S, de Madrigal M, Burciaga A, Martínez ME, Johnson MJ (2016). Technology-assisted stroke rehabilitation in Mexico: a pilot randomized trial comparing traditional therapy to circuit training in a Robot/technology-assisted therapy gym. J NeuroEng Rehabil.

[5] CADTH, 2020. Guidelines for the Economic Evaluation of Health Technologies: Canada | CADTH [WWW Document]. URL https://www.cadth.ca/guidelines-economic-evaluation-health-technologies-canada-0. Accessed 31 Jan 2024.

[6] Damiano DL, Longo E, Carolina de Campos A, Forssberg H, Rauch A. Systematic Review of Clinical Guidelines Related to Care of Individuals With Cerebral Palsy as Part of the World Health Organization Efforts to Develop a Global Package of Interventions for Rehabilitation. Archives of Physical Medicine and Rehabilitation. 2021;102:1764–74. 10.1016/j.apmr.2020.11.015 10.1016/j.apmr.2020.11.015 PMC9619294 33453191

[7] Doran CM (2008). Economic Evaluation of Interventions to Treat Opiate Dependence. Pharmacoeconomics.

[8] Drummond MF. Methods for the economic evaluation of health care programmes, Fourth edition. Oxford, New York: Oxford medical publications, Oxford University Press; 2015.

[9] Fernandez-Garcia C, Ternent L, Homer TM, Rodgers H, Bosomworth H, Shaw L, Aird L, Andole S, Cohen D, Dawson J, Finch T, Ford G, Francis R, Hogg S, Hughes N, Krebs HI, Price C, Turner D, Van Wijck F, Wilkes S, Wilson N, Vale L. Economic evaluation of robot-assisted training versus an enhanced upper limb therapy programme or usual care for patients with moderate or severe upper limb functional limitation due to stroke: results from the RATULS randomized controlled trial. BMJ Open. 2021;11:e042081. 10.1136/bmjopen-2020-042081. 10.1136/bmjopen-2020-042081 PMC8154983 34035087

[10] Gao L, Sheppard L, Wu O, Churilov L, Mohebbi M, Collier J, Bernhardt J, Ellery F, Dewey H, Moodie M, AVERT Trial Collaboration Group. Economic evaluation of a phase III international randomized controlled trial of very early mobilization after stroke (AVERT). BMJ Open. 2019;9:e026230. 10.1136/bmjopen-2018-026230. 10.1136/bmjopen-2018-026230 PMC6537993 31118178

[11] Gomersall JS, Jadotte YT, Xue Y, Lockwood S, Riddle D, Preda A (2015). Conducting systematic reviews of economic evaluations. Int J Evid Based Healthc.

[12] HAS (2020). Choix méthodologiques pour l’évaluation économique à la Haute Autorité de Santé.

[13] Hesse S, Heß A, Werner CC, Kabbert N, Buschfort R (2014). Effect on arm function and cost of robot-assisted group therapy in subacute patients with stroke and a moderately to severely affected arm: a randomized controlled trial. Clin Rehabil.

[14] HIQA. Guidelines for the Economic Evaluation of Health Technologies in Ireland. Health Information and Quality Authority. Dublin: HIQA; 2020. https://www.hiqa.ie/sites/default/files/2020-09/HTA-Economic-Guidelines-2020.pdf.

[15] Hornby TG, Rafferty MR, Pinto D, French D, Jordan N (2022). Cost-Effectiveness of High-intensity Training vs Conventional Therapy for Individuals with Subacute Stroke. Arch Phys Med Rehabil.

[16] Housley SN, Garlow AR, Ducote K, Howard A, Thomas T, Wu D, Richards K, Butler AJ (2016). Increasing Access to Cost Effective Home-Based Rehabilitation for Rural Veteran Stroke Survivors. Austin J Cerebrovasc Dis Stroke.

[17] Husereau D, Drummond M, Augustovski F, de Bekker-Grob E, Briggs AH, Carswell C, Caulley L, Chaiyakunapruk N, Greenberg D, Loder E, Mauskopf J, Mullins CD, Petrou S, Pwu R-F, Staniszewska S, CHEERS 2022 ISPOR Good Research Practices Task Force. Consolidated Health Economic Evaluation Reporting Standards 2022 (CHEERS 2022) Statement: Updated Reporting Guidance for Health Economic Evaluations. Value Health. 2022;25:3–9. 10.1016/j.jval.2021.11.1351. 10.1016/j.jval.2021.11.1351 35031096

[18] Islam MK, Brunner I (2019). Cost-Analysis of virtual reality training based on the Virtual Reality for Upper Extremity in Subacute stroke (VIRTUES) trial. Int J Technol Assess Health Care.

[19] Joseph C, Brodin N, Leavy B, Hagströmer M, Löfgren N, Franzén E (2019). Cost-effectiveness of the HiBalance training program for elderly with Parkinson’s disease: analysis of data from a randomized controlled trial. Clin Rehabil.

[20] Kairy D, Lehoux P, Vincent C, Visintin M (2009). A systematic review of clinical outcomes, clinical process, healthcare utilization and costs associated with telerehabilitation. Disabil Rehabil.

[21] Klobucká S, Klobucký R, Valovičová K, Šiarnik P, Kollár B (2023). Cost-effectiveness analysis of robot-assisted gait training in patients with bilateral spastic cerebral palsy. Cost Effective Resour Allocation.

[22] Lloréns R, Noé E, Colomer C, Alcañiz M (2015). Effectiveness, usability, and cost-benefit of a virtual reality-based telerehabilitation program for balance recovery after stroke: a randomized controlled trial. Arch Phys Med Rehabil.

[23] Lopes RP, Barroso B, Deusdado L, Novo A, Guimarães M, Teixeira JP, Leitão P. Digital Technologies for Innovative Mental Health Rehabilitation. Electronics. 2021;10:2260. 10.3390/electronics10182260.

[24] Lo Kenneth, Stephenson M, Lockwood C (2019). The economic cost of robotic rehabilitation for adult stroke patients: a systematic review. JBI Database System Rev Implement Rep.

[25] Morii Y, Abiko K, Osanai T, Takami J, Tanikawa T, Fujiwara K, Houkin K, Ogasawara K (2023). Cost-effectiveness of seven-days-per-week rehabilitation schedule for acute stroke patients. Cost Effective Resour Allocation.

[26] NICE, 2021. Economic evaluation: health economic studies. The National Institute for Health and Care Excellence (NICE) [WWW Document]. GOV.UK. URL https://www.gov.uk/guidance/economic-evaluation-health-economic-studies. Accessed 31 Jan 2024.

[27] Nixon J, Khan KS, Kleijnen J (2001). Summarizing economic evaluations in systematic reviews: a new approach. BMJ.

[28] Ouzzani M, Hammady H, Fedorowicz Z, Elmagarmid A. Rayyan—a web and mobile app for systematic reviews | Systematic Reviews | Full Text [WWW Document]. https://systematicreviewsjournal.biomedcentral.com/articles/10.1186/s13643-016-0384-4. Accessed 23 May 2023. 10.1186/s13643-016-0384-4 PMC5139140 27919275

[29] Page MJ, McKenzie JE, Bossuyt PM, Boutron I, Hoffmann TC, Mulrow CD, Shamseer L, Tetzlaff JM, Akl EA, Brennan SE, Chou R, Glanville J, Grimshaw JM, Hróbjartsson A, Lalu MM, Li T, Loder EW, Mayo-Wilson E, McDonald S, McGuinness LA, Stewart LA, Thomas J, Tricco AC, Welch VA, Whiting P, Moher D. The PRISMA 2020 statement: an updated guideline for reporting systematic reviews. BMJ. 2021;372:n71. 10.1136/bmj.n71. 10.1136/bmj.n71 PMC8005924 33782057

[30] Pinto D, Heinemann AW, Chang S-H, Charlifue S, Field-Fote EC, Furbish CL, Jayaraman A, Tefertiller C, Taylor HB, French DD (2023). Cost-effectiveness analysis of overground robotic training versus conventional locomotor training in people with spinal cord injury. J Neuroeng Rehabil.

[31] Prvu Bettger J, Green CL, Holmes DN, Chokshi A, Mather RC, Hoch BT, de Leon AJ, Aluisio F, Seyler TM, Del Gaizo DJ, Chiavetta J, Webb L, Miller V, Smith JM, Peterson ED. Effects of Virtual Exercise Rehabilitation In-Home Therapy Compared with Traditional Care After Total Knee Arthroplasty: VERITAS, a Randomized Controlled Trial. J Bone Joint Surg Am. 2020;102:101–9. 10.2106/JBJS.19.00695. 10.2106/JBJS.19.00695 31743238

[32] Rémy-Néris O, Le Jeannic A, Dion A, Médée B, Nowak E, Poiroux É, Durand-Zaleski I, REM Investigative Team*. Additional, Mechanized Upper Limb Self-Rehabilitation in Patients With Subacute Stroke : The REM-AVC Randomized Trial. Stroke. 2021;52:1938–47. 10.1161/STROKEAHA.120.032545. 10.1161/STROKEAHA.120.032545 33910364

[33] Renfrew LM, Paul L, McFadyen A, Rafferty D, Moseley O, Lord AC, Bowers R, Mattison P. The clinical- and cost-effectiveness of functional electrical stimulation and ankle-foot orthoses for foot drop in Multiple Sclerosis: a multicenter randomized trial. Clin Rehabil. 2019;33:1150–62. 10.1177/0269215519842254. 10.1177/0269215519842254 30974955

[34] Stefano M, Patrizia P, Mario A, Ferlini G, Rizzello R, Rosati G. Robotic upper limb rehabilitation after acute stroke by NeReBot: Evaluation of treatment costs. BioMed Research International 2014. 2014. 10.1155/2014/265634 10.1155/2014/265634 PMC4017845 24967345

[35] Tavares E, Coelho J, Rogado P, Correia R, Castro C, Fernandes JB. Barriers to Gait Training among Stroke Survivors : An Integrative Review. J Funct Morphol Kinesiol. 2022;7:85. 10.3390/jfmk7040085. 10.3390/jfmk7040085 PMC9590000 36278746

[36] Wagner TH, Lo AC, Peduzzi P, Bravata DM, Huang GD, Krebs HI, Ringer RJ, Federman DG, Richards LG, Haselkorn JK, Wittenberg GF, Volpe BT, Bever CT, Duncan PW, Siroka A, Guarino PD. An economic analysis of robot-assisted therapy for long-term upper-limb impairment after stroke. Stroke. 2011;42:2630–2. 10.1161/STROKEAHA.110.606442. 10.1161/STROKEAHA.110.606442 PMC4445835 21757677

[37] WHO, 2017. Rehabilitation in health systems. Geneva: World Health Organization; 2017. ISBN-13: 978-92-4-154997-4.

[38] Winstein CJ, Stein J, Arena R, Bates B, Cherney LR, Cramer SC, Deruyter F, Eng JJ, Fisher B, Harvey RL, Lang CE, MacKay-Lyons M, Ottenbacher KJ, Pugh S, Reeves MJ, Richards LG, Stiers W, Zorowitz RD. American Heart Association Stroke Council, Council on Cardiovascular and Stroke Nursing, Council on Clinical Cardiology, and Council on Quality of Care and Outcomes Research, 2016. Guidelines for Adult Stroke Rehabilitation and Recovery: A Guideline for Healthcare Professionals From the American Heart Association/American Stroke Association. Stroke. 2016;47:e98–e169. 10.1161/STR.000000000000009. 10.1161/STR.0000000000000098 27145936

