# Supplementary material for: Economic analysis of digital motor rehabilitation technologies: a systematic review
Source: Health Econ Rev. 2024 Jul 17;14:52. doi: 10.1186/s13561-024-00523-5 (PMC11253330; doi:10.1186/s13561-024-00523-5)

**Appendix 1 : PRISMA checklist**

| **Section and Topic** | **Item #** | **Checklist item** | **Location where item is reported** |
| --- | --- | --- | --- |
| **TITLE** | | |  |
| Title | 1 | Identify the report as a systematic review. | 1 |
| **ABSTRACT** | | |  |
| Abstract | 2 | See the PRISMA 2020 for Abstracts checklist. | 1 |
| **INTRODUCTION** | | |  |
| Rationale | 3 | Describe the rationale for the review in the context of existing knowledge. | 2 |
| Objectives | 4 | Provide an explicit statement of the objective(s) or question(s) the review addresses. | 2 |
| **METHODS** | | |  |
| Eligibility criteria | 5 | Specify the inclusion and exclusion criteria for the review and how studies were grouped for the syntheses. | 3&Appendix 2 |
| Information sources | 6 | Specify all databases, registers, websites, organisations, reference lists and other sources searched or consulted to identify studies. Specify the date when each source was last searched or consulted. | 3& Appendix 3, 4 |
| Search strategy | 7 | Present the full search strategies for all databases, registers and websites, including any filters and limits used. | 4 |
| Selection process | 8 | Specify the methods used to decide whether a study met the inclusion criteria of the review, including how many reviewers screened each record and each report retrieved, whether they worked independently, and if applicable, details of automation tools used in the process. | 4 |
| Data collection process | 9 | Specify the methods used to collect data from reports, including how many reviewers collected data from each report, whether they worked independently, any processes for obtaining or confirming data from study investigators, and if applicable, details of automation tools used in the process. | 4 |
| Data items | 10a | List and define all outcomes for which data were sought. Specify whether all results that were compatible with each outcome domain in each study were sought (e.g. for all measures, time points, analyses), and if not, the methods used to decide which results to collect. | 4 |
|  | 10b | List and define all other variables for which data were sought (e.g. participant and intervention characteristics, funding sources). Describe any assumptions made about any missing or unclear information. | 4 |
| Study risk of bias assessment | 11 | Specify the methods used to assess risk of bias in the included studies, including details of the tool(s) used, how many reviewers assessed each study and whether they worked independently, and if applicable, details of automation tools used in the process. | 5 |
| Effect measures | 12 | Specify for each outcome the effect measure(s) (e.g. risk ratio, mean difference) used in the synthesis or presentation of results. | 6 |
| Synthesis methods | 13a | Describe the processes used to decide which studies were eligible for each synthesis (e.g. tabulating the study intervention characteristics and comparing against the planned groups for each synthesis (item #5)). | 5 |
|  | 13b | Describe any methods required to prepare the data for presentation or synthesis, such as handling of missing summary statistics, or data conversions. | 6 |
|  | 13c | Describe any methods used to tabulate or visually display results of individual studies and syntheses. | 6 |
|  | 13d | Describe any methods used to synthesize results and provide a rationale for the choice(s). If meta-analysis was performed, describe the model(s), method(s) to identify the presence and extent of statistical heterogeneity, and software package(s) used. | 6 |
|  | 13e | Describe any methods used to explore possible causes of heterogeneity among study results (e.g. subgroup analysis, meta-regression). | NA |
|  | 13f | Describe any sensitivity analyses conducted to assess robustness of the synthesized results. | NA |
| Reporting bias assessment | 14 | Describe any methods used to assess risk of bias due to missing results in a synthesis (arising from reporting biases). | NA |
| Certainty assessment | 15 | Describe any methods used to assess certainty (or confidence) in the body of evidence for an outcome. | NA |
| **RESULTS** | | |  |
| Study selection | 16a | Describe the results of the search and selection process, from the number of records identified in the search to the number of studies included in the review, ideally using a flow diagram. | 6 |
|  | 16b | Cite studies that might appear to meet the inclusion criteria, but which were excluded, and explain why they were excluded. | 6 |
| Study characteristics | 17 | Cite each included study and present its characteristics. | 7 |
| Risk of bias in studies | 18 | Present assessments of risk of bias for each included study. | 12 |
| Results of individual studies | 19 | For all outcomes, present, for each study: (a) summary statistics for each group (where appropriate) and (b) an effect estimate and its precision (e.g. confidence/credible interval), ideally using structured tables or plots. | 9 |
| Results of syntheses | 20a | For each synthesis, briefly summarise the characteristics and risk of bias among contributing studies. | 9 |
|  | 20b | Present results of all statistical syntheses conducted. If meta-analysis was done, present for each the summary estimate and its precision (e.g. confidence/credible interval) and measures of statistical heterogeneity. If comparing groups, describe the direction of the effect. | 9 |
|  | 20c | Present results of all investigations of possible causes of heterogeneity among study results. | 17 |
|  | 20d | Present results of all sensitivity analyses conducted to assess the robustness of the synthesized results. | 17, in table 6 |
| Reporting biases | 21 | Present assessments of risk of bias due to missing results (arising from reporting biases) for each synthesis assessed. | NA |
| Certainty of evidence | 22 | Present assessments of certainty (or confidence) in the body of evidence for each outcome assessed. | NA |
| **DISCUSSION** | | |  |
| Discussion | 23a | Provide a general interpretation of the results in the context of other evidence. | 21 |
|  | 23b | Discuss any limitations of the evidence included in the review. | 24 |
|  | 23c | Discuss any limitations of the review processes used. | 24 |
|  | 23d | Discuss implications of the results for practice, policy, and future research. | 24 |
| **OTHER INFORMATION** | | | 3 |
| Registration and protocol | 24a | Provide registration information for the review, including register name and registration number, or state that the review was not registered. | 3 |
|  | 24b | Indicate where the review protocol can be accessed, or state that a protocol was not prepared. | 21 |
|  | 24c | Describe and explain any amendments to information provided at registration or in the protocol. | NA |
| Support | 25 | Describe sources of financial or non-financial support for the review, and the role of the funders or sponsors in the review. | 25 |
| Competing interests | 26 | Declare any competing interests of review authors. | 25 |
| Availability of data, code and other materials | 27 | Report which of the following are publicly available and where they can be found: template data collection forms; data extracted from included studies; data used for all analyses; analytic code; any other materials used in the review. | 25 |

**Appendix 2 : inclusion criteria**

| **Population** | The studies were included if they focused on the motor rehabilitation of patients aged 18 and older, without gender distinction. There is no restriction on pathology; thus, studies involving patients with conditions such as stroke, spinal cord injuries, spinal cord injury patients, patients with lower back pain, pain, multiple sclerosis, traumatic brain injuries, or Parkinson's disease may be included. All phases of the disease, including new patients, those in the acute, subacute, or chronic phases, were considered |
| --- | --- |
| **Intervention** | The review included studies comparing a group of patients undergoing digital technology-assisted rehabilitation to a group undergoing conventional therapy, namely physiotherapy. The types of digital technologies can vary and include technologies such as exoskeletons, video games, treadmills, virtual or augmented reality devices, telerehabilitation devices, sensors, e-health devices, statically assisted body weight treadmills, or bicycles. The intervention group may combine rehabilitation assisted by one or more digital technologies with conventional rehabilitation, while the control group should only adopt conventional therapy |
| **Type of Economic Analysis** | The studies using the following outcome criteria will be included in our review:  Cost Minimization Analysis (CMA) : Assuming equivalence in the effectiveness of rehabilitation between groups, in cost minimization analysis, the objective is to compare the cost of digital technology-assisted rehabilitation to the cost of conventional physiotherapy. The conclusion on efficiency is therefore based solely on cost results. The efficient strategy is the one with lower costs.  Cost-Effectiveness Analysis (CEA) : CEA results are presented in the form of relative costs to obtain one unit of effect from digital technology-assisted rehabilitation compared to conventional rehabilitation. The effect is expressed in physical units (e.g., upper limb score when the Fugl-Meyer scale is used for upper limb evaluation).  Cost-Utility Analysis (CUA) : In CUA, results are presented in the form of relative costs for gaining one unit of utility with digital technology-assisted rehabilitation compared to conventional rehabilitation. In this case, the effect is expressed in quality-adjusted life years (QALY).  Cost-Benefit Analysis (CBA) : Cost and effectiveness results are expressed in monetary units, and the ratio is interpreted as the relative cost to obtain one unit of benefit with the intervention group compared to the control group.  Budget Impact Analysis (BIA) : BIA estimates the potential financial impact of the introduction and dissemination of a digital technology-assisted rehabilitation strategy in relation to the estimated beneficiary population |
| **Design** | Economic studies on digital rehabilitation technologies were included. The economic component of the review considers studies that employed cost minimization analysis, cost-effectiveness analysis, cost-utility analysis, cost-benefit analysis, or budget impact analysis comparing digitally assisted motor rehabilitation to conventional physiotherapy. Partial economic evaluations were also included, contrary to the original plan. To gather empirical data from prospective clinical trials, modeling studies, systematic reviews, and meta-analyses were not included |

**Appendix 3 : Keywords**

| **Themes** | **Keywords** |
| --- | --- |
| Ilness | Impairment OR "disability OR "cerebral palsy" OR "Spinal cord injur*" OR "Parkinson OR "Multiple Sclerosis" OR Sclerosis OR "Brain Injur*" OR "Cerebrovascular Accident" OR Stroke OR "bell palsy" OR "trigeminal neuralgia" OR Meningitis OR pain OR "Alzheimer’s disease" OR "huntington disease" |
| Exercise | Rehabilitation OR Telerehabilitation OR "Exercise therap*" OR Locomotion OR exercise* OR "Physical activity" OR "sports" OR "strength training" OR "resistance training" OR "fitness training" OR "running" OR walk OR "physical therapy" OR "exercise therapy" OR "physical activit*" OR "physical therapy modalities" OR "physiotherap*" OR "yoga" OR "resistance training" OR "aerobic exercise" OR "weight training" OR "strength training" OR "endurance training" OR "bicycling" OR "bicycling" OR "swimming" |
| Technology of rehabilitation | "Digital technology" OR " Virtual reality" OR "Augmented reality" OR gaming OR Treadmill OR "walking aid" OR Orthose OR "Mechanical trainer" OR Sensor OR "Wearable Electronic Devices" OR Tablet OR phone OR Robot* OR Exoskeleton OR "Gait Trainer" |
| Economic evaluation | "cost benefit analysis" OR "cost effectiveness" OR "cost utility" OR "economic evaluation" OR "incremental cost effectiveness ratio" OR "quality adjusted life years" OR "QALY" OR "ICER" OR "economic analysis" OR "economic impact" OR “cost minimization analysis” |

**Appendix 4 : search strategy**

| Base de données | Termes de recherche |
| --- | --- |
| PubMed | ((Impairment[tiab] OR disability[tiab] OR "cerebral palsy"[tiab] OR "cerebral palsy"[Mesh] OR "Spinal cord injury"[tiab] OR "Spinal Cord Injuries"[Mesh] OR Parkinson[tiab] OR "Multiple Sclerosis"[Mesh] OR Sclerosis[tiab] OR "Brain Injury"[tiab] OR "Brain Injuries"[Mesh] OR "Cerebrovascular Accident"[tiab] OR Stroke[tiab] OR Stroke[Mesh] OR "bell palsy"[tiab] OR "bell palsy"[Mesh] OR "trigeminal neuralgia"[tiab] OR "trigeminal neuralgia"[Mesh] OR Meningitis[tiab] OR meningitis[Mesh] OR pain[tiab] OR pain[Mesh] OR "Alzheimer’s disease"[tiab] OR "huntington disease"[tiab] OR "huntington disease"[Mesh]) AND ("Digital technology"[tiab] OR " Virtual reality"[tiab] OR "Virtual Reality"[Mesh] OR "Augmented reality"[tiab] OR "Augmented Reality"[Mesh] OR gaming [tiab] OR Treadmill[tiab] OR " walking aid"[tiab] OR Walking[Mesh] OR Orthose[tiab] OR "Mechanical trainer"[tiab] OR Sensor[tiab ] OR "Wearable Electronic Devices"[Mesh] OR Tablet[tiab] OR Tablet[Mesh] OR phone[tiab] OR Robot*[tiab] OR Robotics[Mesh] OR Exoskeleton [tiab] OR "Gait Trainer"[tiab]) AND (Rehabilitation[tiab] OR Rehabilitation[Mesh] OR Telerehabilitation[tiab] OR Telerehabilitation[Mesh] OR "Exercise therapy" OR "Exercise therapy"[Mesh] OR Locomotion[tiab] OR Locomotion[Mesh])) AND (Economic [tiab] OR Economics [Mesh] OR "Economic evaluation"[tiab] OR "Cost-Benefit analysis"[tiab] OR "Cost-Benefit analysis"[Mesh] OR "Cost effectiveness"[tiab] OR "Cost-utility"[tiab] OR "Cost Analysis"[tiab] OR "Costs and Cost Analysis"[Mesh] OR "Health Care Costs"[tiab] OR "Health Care Costs"[Mesh] OR efficiency[tiab] OR efficiency[Mesh] OR "Incremental cost-effectiveness ratio" OR "adjusted life years" OR "QALY" OR "ICER" OR "economic analysis" OR "economic impact" OR “cost minimization analysis”)  **Filters**: Clinical trials |
| Science Direct | (Disability OR impairment) AND (Rehabilitation) AND ("digital technology" OR Robot OR "Gait trainer") AND ("Cost analysis" OR "Economic evaluation" OR “ICER”)  **Filters:**  Articles of medical sector items |
| Web of science | TS = ((impairment OR disability OR "cerebral palsy" OR "cerebral palsy" OR "Spinal cord injury" OR Parkinson OR "Multiple Sclerosis" OR sclerosis OR "Brain Injury" OR "Cerebrovascular Accident" OR stroke OR "bell palsy" OR "trigeminal neuralgia" OR meningitis OR pain OR "Alzheimer’s disease" OR "Huntington disease") AND ("Digital technology" OR " Virtual reality" OR "Augmented reality" OR gaming OR treadmill OR " walking aid" OR orthose OR "Mechanical trainer" OR "Wearable Electronic Devices" OR tablet OR phone OR robot* OR exoskeleton OR "gait trainer") AND (rehabilitation OR telerehabilitation OR " exercise OR therapy " OR locomotion) AND (Economic OR " economic evaluation " OR "cost-benefit analysis " OR " cost effectiveness" OR " cost-utility " OR "costs analysis" OR "health care costs" OR efficiency OR "Incremental cost-effectiveness ratio" OR "adjusted life year" OR "QALY" OR "ICER" OR "economic analysis" OR "economic impact" OR "cost minimization analysis"))  **Filters:**   - Document types: Articles - Languages: English - Research areas: Rehabilitation |
| Cochrane (CENTRAL) | ("Disability" OR "Impairment" OR "Cerebral Palsy" OR "Spinal Cord Injury" OR "Parkinson's Disease" OR "Multiple Sclerosis" OR "Traumatic Brain Injury" OR "Cerebrovascular accident" OR "Stroke" OR "Bell's Palsy" OR "Trigeminal Neuralgia" OR "Meningitis" OR "Pain" OR "Alzheimer's Disease" ) AND ( "Digital health technology" OR "Virtual Reality" OR "Video-based training" OR "Treadmill" OR "Walking" OR "Orthotic Device " OR "Tablet" OR "Robot" OR "Robotic-assisted Gait Training " OR "Gait Training Procedure" ) AND ("Rehabilitation Therapy" OR "Telerehabilitation" OR "Physical exercise therapy" OR "Locomotor Training" ) AND ( "Costs and Cost Analysis" OR "Healthcare costs" OR "Cost-Benefit Analysis" OR "Incremental cost-effectiveness ratio" OR "QALY" OR "ICER" OR "economic analysis" OR "economic impact" OR “cost minimization analysis”) |
| Scopus | ((Impairment OR disability OR "cerebral palsy" OR "cerebral palsy" OR "Spinal cord injury" OR Parkinson OR "Multiple Sclerosis" OR sclerosis OR "Brain Injury" OR "Cerebrovascular Accident" OR stroke OR "bell palsy" OR "trigeminal neuralgia" OR meningitis OR pain OR "Alzheimer’s disease" OR "Huntington disease") AND ("Digital technology" OR " Virtual reality" OR "Augmented reality" OR gaming OR treadmill OR " walking aid" OR orthose OR "Mechanical trainer" OR "Wearable Electronic Devices" OR tablet OR phone OR robot* OR exoskeleton OR "gait trainer") AND (Rehabilitation OR telerehabilitation OR " exercise OR therapy " OR locomotion) AND (Economic OR " economic evaluation " OR "cost-benefit analysis " OR " cost effectiveness" OR " cost-utility " OR "costs analysis" OR "health care costs" OR efficiency OR "Incremental cost-effectiveness ratio" OR "adjusted life year" OR "QALY" OR "ICER" OR "economic analysis" OR "economic impact" OR "cost minimization analysis"))  **Filters:**   - Document types: Articles - Subject areas: medicine - Exact keywords: Rehabilitation |

**Appendix 5 :** **Study characteristics**

| **Location** | USA | | | UK | | France | Germany | Italia | Spain | Mexico | BE, DK and NO |  |
| --- | --- | --- | --- | --- | --- | --- | --- | --- | --- | --- | --- | --- |
|  |  |  |  |  |  |  |  |  |  |  |  |  |
| **Ilness** | AVC | | | | | | | | | | Knee Arthroplasty | |
|  |  |  |  |  |  |  |  |  |  |  |  |  |
| **Design** | RCT | | | | | | | | | | | |
|  |  |  |  |  |  |  |  |  |  |  |  |  |
| **Economic evaluation** | Cost-utility analysis (CUA) | | | | Cost minimization analysis (CMA) | | | | | | | |
|  |  |  |  |  |  |  |  |  |  |  |  |  |
| **ICER Estimation** | YES | | | | NO | | | | | | | |
|  |  |  |  |  |  |  |  |  |  |  |  |  |
| **Discount rate** | YES | | NO | | | | | | | | | |
|  |  |  |  |  |  |  |  |  |  |  |  |  |
| **Sensitivity analysis** | YES | | | NO | | | | | | | | |
|  |  |  |  |  |  |  |  |  |  |  |  |  |
| **0%** | **9%** | **18%** | **27%** | **36%** | **45%** | **55%** | **64%** | **73%** | **82%** | **91%** | **100%** |  |

**BE :** Belgium **; DK :** Danemark **; NO :** Norway

**Appendix 6 : Reported health service resource use**

| Author | Reported health service resource use |
| --- | --- |
| Wagner et al., 2011 | 1. **the capital cost of the robot**: costs of acquisition, maintenance, and usage of robots used for robot-assisted therapy 2. **Healthcare professionals' costs**: fees of doctors, physiotherapists, occupational therapists involved in patient care 3. **Medical consultation costs**: expenses related to medical consultations to assess and monitor patients. 4. **Follow-up examination costs**: costs of medical examinations and follow-up tests to evaluate patients' progress 5. **Medication costs**: costs of prescribed medications in the therapy process 6. **Additional medical equipment costs**: costs of specific equipment used in robot-assisted therapy 7. **Hospital resource costs**: costs associated with the use of hospital facilities for therapy sessions 8. **Travel fees** |
| Hesse et al.,2014 | 1. **Costs of Medical and paramedical staff** 2. **Infrastructure and equipment**: Costs related to the use of therapeutic robots, rehabilitation equipment, facilities, and medical supplies 3. **Consumable materials**: Medical supplies used during therapy sessions 4. **Training fees**: Costs related to training the staff involved in robotic therapy |
| Stefano et al., 2014 | 1. **Robot’s cost**: acquisition, maintenance, and operation costs of the robots used for upper limb rehabilitation. 2. **Healthcare professionals' costs**: fees of physicians, physiotherapists, occupational therapists 3. **Medical consultation costs** 4. **Follow-up examination costs** 5. **Medication costs** 6. **Hospital resource costs** |
| Lloréns et al., 2016 | 1. **Costs of virtual reality technology** 2. **Healthcare professionals' costs** 3. **Monitoring and support costs** 4. **Training and education costs** |
| Bustamante Valles et al., 2016 | 1. **Costs of usual therapy**: expenses related to traditional stroke rehabilitation therapy sessions. 2. **Costs of circuit training in a robot/technology-assisted therapy gym** 3. **Healthcare professionals' costs** 4. **Equipment costs** 5. **Medication costs** 6. **Administrative and overhead costs** |
| Housley et al., 2016 | 1. **Healthcare professionals' cost** 2. **Rehabilitation equipment costs** 3. **Telehealth costs**: expenses related to setting up telehealth solutions for remote supervision and monitoring of patients 4. **Home visit costs** |
| Adie et al., 2017 | 1. **Nintendo Wii^TM^ ‘s cost (acquisition costs of the Wii console, controllers)** 2. **Healthcare professionals' fees** 3. **Personnel training costs, monitoring and evaluation expenses for participants' progress** |
| Islam and Brunner, 2019 | 1. Therapist salary 2. Cost of YouGrabber (now Bi-Manu-Trainer): |
| Prvu Bettger et al., 2020 | 1. **Health service use costs** 2. **Home health physical therapy costs** 3. **Outpatient physical therapy costs** 4. **Physician clinic costs** 5. **Rehospitalization costs** |
| Rémy-Néris et al., 2021 | 1. **Costs of mechanized upper limb self-rehabilitation devices**: costs of acquiring, maintaining, and using the mechanized devices used for upper limb rehabilitation 2. **Healthcare professionals' costs** 3. **Medical consultation costs** 4. **Follow-up examination costs** 5. **Medication costs** 6. **Hospital resource costs** |
| Fernandez-Garcia et al., 2021 | 1. **Robot cost** 2. **Visits to emergency services** 3. **Outpatient appointments** 4. **Hospital stays** 5. **General practitioner and nursing services** 6. **Therapy services costs** 7. **Medications** 8. **Community healthcare** 9. **Social services** 10. **Stays in residential and nursing homes** |

**Appendix 7 : Quality scores of included studies**

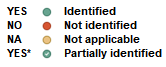

Supplement: Supplementary file 1 — Supplementary Material 1. [file 13561_2024_523_MOESM1_ESM.docx]
